# Construction of Zinc Oxide into Different Morphological Structures to Be Utilized as Antimicrobial Agent against Multidrug Resistant Bacteria

**DOI:** 10.1155/2015/536854

**Published:** 2015-09-16

**Authors:** M. F. Elkady, H. Shokry Hassan, Elsayed E. Hafez, Ahmed Fouad

**Affiliations:** ^1^Fabrication Technology Department, Advanced Technology and New Materials Research Institute (ATNMRI), City of Scientific Research and Technological Applications, Alexandria 21934, Egypt; ^2^Chemical and Petrochemical Engineering Department, Egypt-Japan University of Science and Technology, New Borg El-Arab City, Alexandria 21934, Egypt; ^3^Electronic Materials Research Department, Advanced Technology and New Materials Research Institute (ATNMRI), City of Scientific Research and Technological Applications, Alexandria 21934, Egypt; ^4^Plant Protection and Biomolecular Diagnosis Department, Arid Lands Cultivation Research Institute, City of Scientific Research and Technological Applications, New Borg El-Arab City, Alexandria 21934, Egypt

## Abstract

Nano-ZnO has been successfully implemented in particles, rods, and tubes nanostructures via sol-gel and hydrothermal techniques. The variation of the different preparation parameters such as reaction temperature, time, and stabilizer agents was optimized to attain different morphological structures. The influence of the microwave annealing process on ZnO crystallinity, surface area, and morphological structure was monitored using XRD, BET, and SEM techniques, respectively. The antimicrobial activity of zinc oxide produced in nanotubes structure was examined against four different multidrug resistant bacteria: Gram-positive (*Staphylococcus aureus* and *Bacillus subtilis*) and Gram-negative (*Escherichia coli* and *Pseudomonas aeruginosa*) strains. The activity of produced nano-ZnO was determined by disc diffusion technique and the results revealed that ZnO nanotubes recorded high activity against the studied strains due to their high surface area equivalent to 17.8 m^2^/g. The minimum inhibitory concentration (MIC) of ZnO nanotubes showed that the low concentrations of ZnO nanotubes could be a substitution for the commercial antibiotics when approached in suitable formula. Although the annealing process of ZnO improves the degree of material crystallinity, however, it declines its surface area and consequently its antimicrobial activity.

## 1. Introduction

Nanotechnology offers unique approaches to control a wide variety of biological and medical processes that occur at nanometer length and it is believed to have a successful impact on biology and medicine [1].

In this world of emerging nanotechnology, one of the primary concerns is the potential environment impact of nanoparticles (NPs) [2]. An efficient way to estimate nanotoxicity is to monitor the response of bacteria exposed to these particles. Resistance of bacteria to bactericides and antibiotics has increased in recent years due to the development of resistant strains. Some antimicrobial agents are extremely irritant and toxic and there is much interest in finding ways to formulate new types of safe and cost-effective biocidal materials [3, 4]. Previous studies have shown that antimicrobial formulations in the form of nanoparticles could be used as effective bactericidal materials [5, 6]. Recently, it has been demonstrated that highly reactive metal oxide nanoparticles exhibit excellent biocidal action against Gram-positive and Gram-negative bacteria [7]. Thus, the preparation, characterization, surface modification, and functionalization of nanosized inorganic particles open the possibility of formulation of a new generation of bactericidal materials [8].

Application of nano-zinc oxide in food systems may be effective at inhibiting certain food-borne pathogens. It was reported that nano-zinc oxide possesses strong antimicrobial activity against* Listeria monocytogenes*,* Salmonella enteritidis*, and* Escherichia coli O*_*157*_ *:H*_*7*_ [9]. Moreover, nano-zinc oxide has a good potential to be coated on a plastic film to make antimicrobial packaging against bacteria such as* Escherichia coli* and* Staphylococcus aureus* [10].

By controlling the structure precisely at nanoscale dimensions, one can control and modify its surface layer for enhanced aqueous solubility, biocompatibility, or bioconjugation; nanoparticles exhibit attractive properties like high stability and the ability to modify their surface characteristics easily [11].

Sol-gel and hydrothermal methods have been considered to be the most attractive nanomaterials fabrication tools in the recent years, and they have an edge over all other processing methods due to their reliable control of the shape and size of the nano-zinc oxide without requiring the expensive and complex equipment.

The hydrothermal technique occupies a unique place owing to its advantages over conventional technologies. The hydrothermal processing of advanced materials has lots of advantages and can be used to give high product purity and homogeneity, crystal symmetry, metastable compounds with unique properties, narrow particle size distributions, a lower sintering temperature, a wide range of chemical compositions, single-step processes, dense sintered powders, submicron particles to nanoparticles with a narrow size distribution using simple equipment, lower energy requirements, fast reaction times, and the lowest residence time, as well as for the growth of crystals with polymorphic modifications, for the growth of crystals with low to ultralow solubility, and as a host of other applications [12].

The sol-gel process can shortly be defined as the conversion of a precursor solution into an inorganic solid via inorganic polymerization reactions induced by water. In general, the precursor or starting compound is either an inorganic (no carbon) metal salt (chloride, nitrate, sulphate, acetate, etc.) or a metal organic compound such as an alkoxide. Metal alkoxides are the most widely used precursors, because they react readily with water and are known for many metals. Some alkoxides, which are widely used in industry, are commercially available at low cost (Zn, Si, Ti, Al, and Zr), whereas other ones are hardly available, or only available at very high costs (Mn, Fe, Co, Ni, Cu, Y, Nb, and Ta). In general, the preparation of nano-zinc oxide from zinc acetate using sol-gel processing is one of the most successful techniques, which produced nanostructures of the best crystalline quality [13–15].

The objective of this investigation is to synthesize nano-zinc oxide with different morphological structures using hydrothermal and sol-gel techniques to investigate the antimicrobial activity of the most proper synthesized nano-zinc oxide that attains the highest surface area against Gram-positive and Gram-negative bacteria. The influence of the annealing process using Microwave Assisted Technology (MAT) on the antimicrobial efficiency of the most proper prepared nano-zinc oxide will be assigned.

## 2. Materials and Methods

### 2.1. Synthesis of Nano-Zinc Oxide via Sol-Gel and Hydrothermal Techniques

Zinc oxide nanopowder with different morphological structures was synthesized via sol-gel and hydrothermal techniques. The variation of the preparation conditions was optimized in order to attain ZnO nanopowder with high surface area. 14 mM aqueous solution of zinc acetate dihydrate was mixed with 0.25 mM of different studied surfactants and the solution pH was adjusted at 9 using 50 mM of sodium hydroxide. The resulting reactant solution mixture was heated at various temperatures on agitated hot plate for the sol-gel technique. Regarding the hydrothermal preparation technique, the solution reaction mixture was aged in an autoclave at 50 Kpsi for different reaction time intervals. The resulting ZnO white powders were washed several times with distilled water and absolute ethanol to remove any residual salts and centrifuged at 6000 rpm for 30 minutes to separate the nano-zinc oxide. Finally, the resultant powders were dried at 60°C under air atmosphere overnight. The different parameters experiments were carried out to determine the optimum conditions for zinc oxide formation. The different preparation parameters such as the reaction temperatures (60, 70, 80, and 90°C), reaction time (3, 6, 12, 24, and 48 hours), and surfactant types (PVA, PVP, CTAB, PEG, and TEA) were optimized to attain zinc oxide with different morphological nanostructures.

### 2.2. Annealing of Prepared ZnO Nanopowders

Annealing process is known to affect the extent of material crystallization [16]. The most proper morphological nanostructured zinc oxide (nanotube) will be annealed at three different temperatures, 500, 700, and 900°C, for one hour using Microwave Assisted Technology (MAT). The annealed samples were then allowed to cool down naturally back to room temperature prior to characterization. The influence of annealing temperature on zinc oxide crystallinity and morphological structure was examined. The antimicrobial efficiency of the prepared ZnO in nanotube morphology after and before annealing process was compared using one type of Gram-positive and another of Gram-negative bacteria isolate.

### 2.3. Characterization of ZnO Nanopowders

The physical properties of the synthesized nano-zinc oxide with different morphological structures were investigated using different techniques. The morphological structure and the chemical compositions of the prepared ZnO nanopowder and annealed samples were examined.

#### 2.3.1. X-Ray Diffraction Analysis (XRD)

X-ray powder diffractometry was carried out using (Shimadzu 7000) diffractometer with Cu K_*α*_ radiation beam (*λ* = 0.154060 nm) to determine the crystalline structure of the different prepared ZnO nanopowders. The finely powdered samples of the nano-zinc oxide were packed into a flat aluminum sample holder, where the X-ray source was a rotating anode operating at 30 kV and 30 mA with a copper target. Data were collected between 10° and 80° in 2*θ*.

#### 2.3.2. Scanning Electron Microscopy (SEM)

The scanning electron microscope is based on scanning a finely focused electron beam across the surface of a specimen. The reflected signals are collected, and their intensities are displayed on a cathode-ray-tube screen by brightness modulation. The ease of sample imaging using scanning electron microscope (JEOL JSM 6360LA, Japan) over large distances is quite appealing. The SEM is also extensively employed for the generation of dimensional and spatial relationship details of structure elements. The surface of the prepared and annealed ZnO nanopowders was scanned to investigate the homogeneity of the nanopowder and to measure the dimensional structures of the different prepared powders' architectural morphology.

#### 2.3.3. Surface Area (BET)

In order to monitor the effect of annealing process on the zinc oxide surface area and subsequently on its antimicrobial activity, the average pore diameter and specific surface area BET (Brunauer-Emmett-Teller) of the most proper prepared ZnO in nanotube morphological structure before and after annealing process were measured on a Quantachrome NOVA 1000 (Boynton Beach, FL, USA) in nitrogen atmosphere.

### 2.4. Bacterial Isolates

The purified bacterial isolates, Gram-positive bacteria (*Staphylococcus aureus ATCC 29213* and* Bacillus subtilis ATCC 23857*) and Gram-negative bacteria (*Escherichia coli ATCC 25922* and* Pseudomonas aeruginosa ATCC 27853*), were kindly provided by Professor Elsayed Elsayed Hafez (Plant Protection and Biomolecular Diagnosis Department, Arid Lands Cultivation Research Institute, City of Scientific Research and Technological Applications (SRTA City)). All the bacterial isolates were grown and maintained on nutrient broth.

### 2.5. Antimicrobial Procedure

Antimicrobial bioassay was carried out using zinc oxide prepared in nanotubes structure before and after annealing against different bacterial isolates, Gram-positive bacteria (*Staphylococcus aureus* and* Bacillus subtilis*) and Gram-negative bacteria (*Escherichia coli* and* Pseudomonas aeruginosa*), using agar diffusion technique in sterilized Petri dishes on the media suitable for tested organisms [17]. Clean Petri dishes containing the media suitable for the growth of the tested pathogens were prepared and inoculated with the tested organisms. Using sterilized cork borer, wells were made in the media. Some wells were inoculated with 100 *μ*L of 10 and 30 mg for each nano-zinc oxide suspension sample. All Petri dishes were incubated at conditions as illustrated before [18]. Then, plates were incubated at 37°C for 24 h. The measured inhibition zones were considered as bactericidal activity of the examined ZnO concentration. The minimum inhibitory concentration (MIC) of zinc oxide was demonstrated for each strain isolate in separate manner.

## 3. Results and Discussion

### 3.1. Synthesis and Characterization of Nano-Zinc Oxide with Different Morphological Structures via Sol-Gel Technique

In order to attain different morphological nanostructures from the synthesized zinc oxide using two different techniques, the variation impact of preparation parameters for zinc oxide production will be examined. Sol-gel technique was utilized for nano-zinc oxide production in the presence of different surfactant agents. The crystalline and morphological structures of the different produced materials were screened using XRD and SEM, respectively.

#### 3.1.1. Synthesis of Nano-Zinc Oxide in the Presence of Surfactant

*(1) Mechanism of Nano-Zinc Oxide Formation in the Presence of Surfactant.* In an aqueous solution, metal cations M^+*Z*^ are solvated by water giving rise to aqua ions, typically [M(OH_2_)_*n*_]^+*Z*^. The M–OH_2_ bond is polarized which facilitates deprotonation of the coordinated water. In dilute solutions, a range of monomeric species exist such as ([M(OH_2_)_*n*−*p*_(OH)_*p*_]^+(*z* − *p*)^) and other hydroxyl species [M(OH)_*n*_]; ultimately oxygen ions are formed. In order to form the polynuclear species, which subsequently develop into metal oxide particles, reactions involving condensation reactions must occur. Two important processes have been recognized; olation is the formation of an “ol” bridge by reaction of a hydroxo- and aquo species as follows [19]:

(1) $$\begin{array}{c} M\text{–}OH+M\text{–}OH_{2}⟶M\text{–}OH\text{–}M+H_{2}O \end{array}$$

Oxolation leads to an “oxo” bridge by the dehydration of hydroxospecies:

(2) $$\begin{array}{c} M_{2}\text{–}{\left(\;OH\;\right)}_{2}⟶M\text{–}O\text{–}M+H_{2}O \end{array}$$

Zinc hydroxide is amphoteric and complexation by OH^−^ can lead to soluble species such as “[Zn(OH)_3_]^−^” and “[Zn(OH)_4_]^−2^” and hence “zinc hydroxide” is more soluble in basic solution [20].

**Figure 1:**
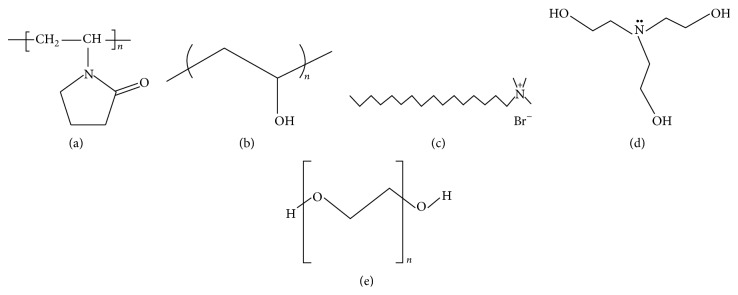
Chemical structure of (a) PVP, (b) PVA, (c) CTAB, (d) TEA, and (e) PEG.

*(A) Effect of Surfactant Type on Both the Crystalline and Morphological Structures of Prepared ZnO.* The surfactant should be characterized by its alkaline nature. Based on this concept, polyvinylpyrrolidone (PVP), polyvinyl alcohol (PVA), cetyltrimethylammonium bromide (CTAB), triethanolamine (TEA), and polyethylene glycol (PEG) were selected as surfactant agents where they contain base radical in their structures as investigated in Figure 1. This figure demonstrated that these surfactants may act as a weak base due to their single lone pair of electrons attached on either nitrogen or oxygen atoms inside their structures and used as complexing agents with the metals, leading to dissolution of zinc hydroxide into zinc oxide [21, 22].

**Figure 2:**
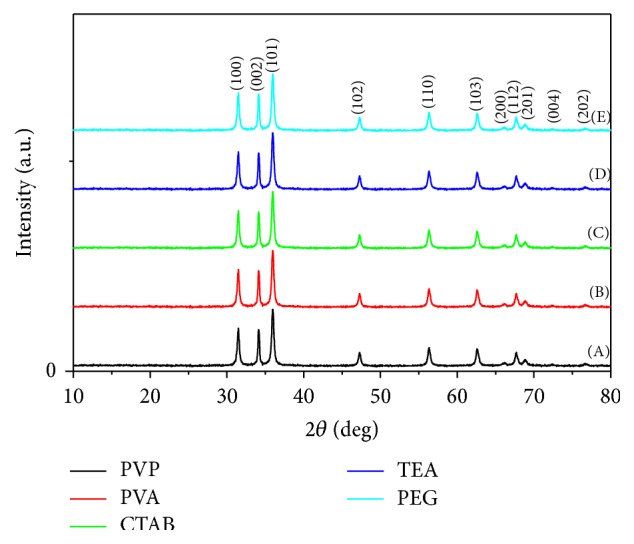
XRD patterns for ZnO nanopowders via sol-gel technique with different surfactant agents: (A) PVP, (B) PVA, (C) CTAB, (D) TEA, and (E) PEG.

*(i) Crystalline Structure (XRD).* The X-ray diffraction patterns of the nanocrystalline phases of the prepared zinc oxide nanopowder using different surfactant agents were compared with the zinc oxide wurtzite phase (JCPDS card number 01-089-1397). It was indicated from Figure 2 that all the prepared ZnO samples using the different surfactant agents showed wurtzite crystal structure with high intensity. Figure 2 illustrates both the 2*θ* values for reference wurtzite phase (JCPDS card number 01-089-1397; *a* = 0.3253 nm, *c* = 0.5213 nm, and *u* = 1.6025) and the measured 2*θ* values for the different synthesized ZnO nanopowders in the presence of different surfactants. The sharp peaks indicate that the products are well crystallized and oriented [23]. All the diffraction peaks can be well indexed to the hexagonal phase of ZnO reported in JCPDS card number 01-089-1397 and no other characteristic peaks are observed [24, 25].

**Figure 3:**
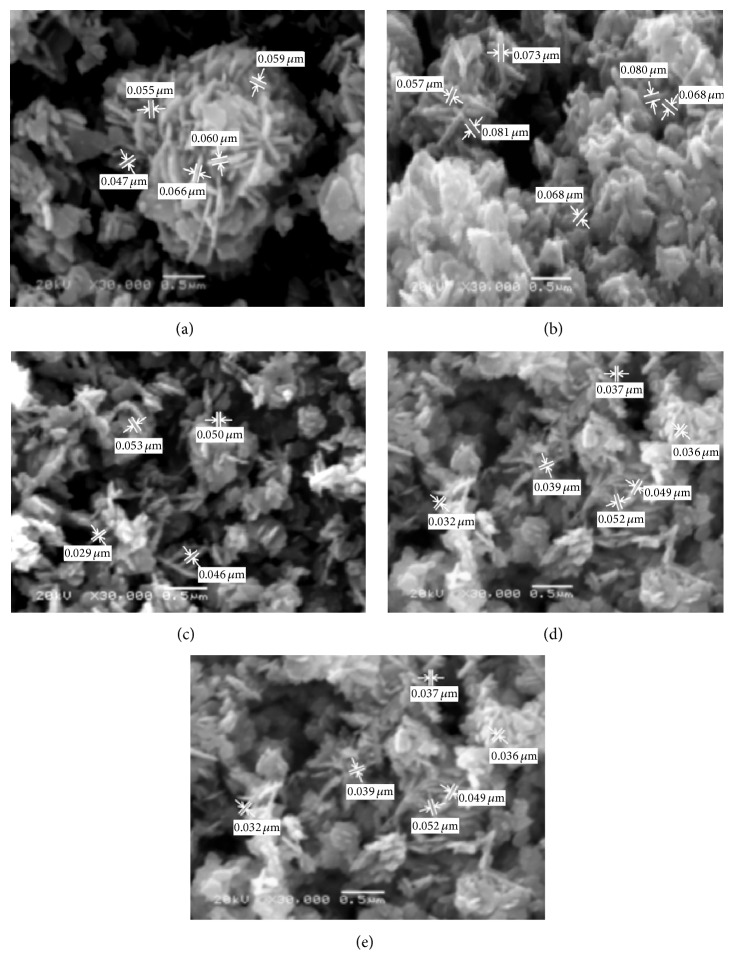
SEM micrographs of ZnO prepared using sol-gel technique with different surfactant agents: (a) PVP, (b) PVA, (c) CTAB, (d) TEA, and (e) PEG.

**Figure 4:**
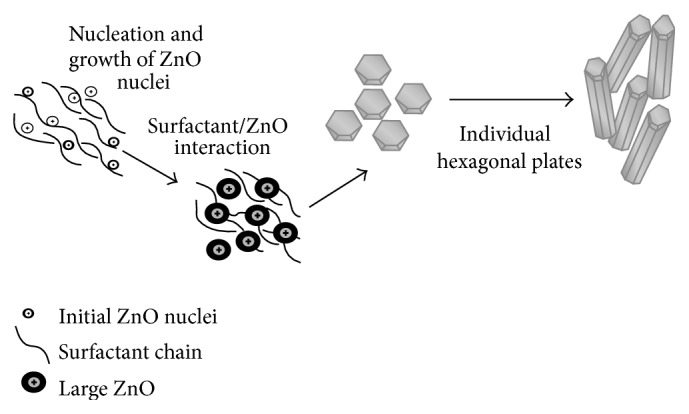
Schematic representation of ZnO nanorods formation mechanism.

*(ii) Morphological Structure (SEM).* Figure 3 investigated the morphological structures of the different prepared ZnO in the presence of different surfactants. The formation of different nanorods and nanoparticles that possess well-defined hexagonal faces was indicated for the different studied surfactants. In case of using PVP and PVA as surfactants, a large quantity of straight rods and a small amount of irregular ones are formed. It was observed that the aspect ratio (the average length-to-diameter ratio) of formed nanorods decreased from 9 to 4 with the surfactant change from PVP to PVA. The suggested mechanism for nanorods ZnO formation in the presence of either PVP or PVA may be considered due to the decomposition of Zn(OH)_2_ within 60 minutes' heating that formed ZnO nucleation. The remaining reaction time may be considered as the time of nuclei growth in other directions that resulted from dissolution and reprecipitation of the existing ZnO particles that formed the nanorods structures. On the other hand, it was evident from Figure 3 that ZnO formed in nanoparticles structure with lower diameters ranged from 30 to 50 nm in the presence of CTAB, TEA, and PEG as surfactants. Accordingly, all the reaction time for ZnO formation in case of utilizing CTAB, TEA, and PEG as surfactants may be spent for decomposition of Zn(OH)_2_ to form ZnO nucleation that was promoted into crystalline structures of nanoparticles at the same nucleation direction. So, it is concluded that the surfactant type affects the size and morphology of ZnO formed according to the nucleation and growth mechanisms [26]. It is indispensable to understand the growth mechanism in order to control and design tailored structures. In the ZnO and surfactant system, the chemical adsorption is basically assigned to the coordination between the surfactant and Zn^2+^ ions or ZnO. Generally, surfactants were used as capping agent because there is a strong interaction between the surfaces of nanocrystals and surfactant based on the strong coordination ability of O and N atoms in the surfactant. It is believed that the selective adsorption of surfactants on various crystallographic planes of the nanocrystals played a vital role in controlling the morphology of the products [27]. Based on the degree of adsorption, the electrostatic force of attraction, and interaction between the surfactant molecules and Zn^2+^ ionic group, a particular morphology with specific orientation is synthesized [28], where the surfactant in aqueous solution formed a microreactor through various interactions. When reactants were added, the precursors of ZnO nucleated in this reactor [29]. Subsequently, the reactor could significantly prevent the growth of some special crystalline faces and also preferentially promoted other crystalline faces' growth (Figure 4).

Furthermore, by coordinating the water from the dehydration reaction of the ZnO precursor, surfactant may accelerate ZnO nucleation and promote ZnO crystallization [29]. So, it is possible to realize that the nucleation and growth of ZnO can be also controlled by the coordination between surfactant and water [30]. Concerning the PVP and PVA behaviors for ZnO formation, it was observed that the density of ZnO rods increases. Accordingly, it may be predicted that the surfactant accelerates the nucleation of ZnO [31]. So, PVP and PVA produced nanorods structure where CTAB, PEG, and TEA produced nanoparticles. Accordingly, PVP was selected as the optimum surfactant that produces ZnO in nanorods structures.

*(B) Effect of Reaction Temperature on Both the Crystalline and Morphological Structures of Prepared ZnO.* The temperature influenced morphology evolution and its effect on physicochemical properties of synthesized nano-zinc oxide. So, simple study on the relationship between the reaction temperature and the morphological structures of synthesized nano-zinc oxide will be examined. The nucleation and growth mechanisms for different ZnO architectural structure will be discussed according to their characterizations.

**Figure 5:**
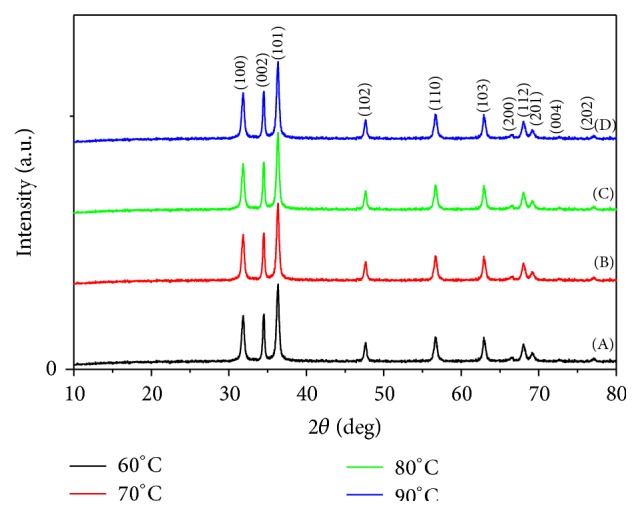
XRD patterns for ZnO nanopowders via sol-gel technique at different reaction temperatures: (A) 60°C, (B) 70°C, (C) 80°C, and (D) 90°C.

*(i) Crystalline Structure (XRD).* The standard XRD patterns of the different produced ZnO are used for relative comparison of crystal structures for the ZnO nanopowders, prepared at different reaction temperatures.  Figure 5 showed that the as-synthesized ZnO nanopowders produced diffraction patterns and they are well indexed as crystalline hexagonal phase wurtzite structure which can be indexed with the zinc oxide wurtzite phase (JCPDS card number 01-089-1397). No peak attributable to possible impurities is observed. The sharp diffraction peaks signify that the as-prepared ZnO nanostructures have high crystallinity [32].

**Figure 6:**
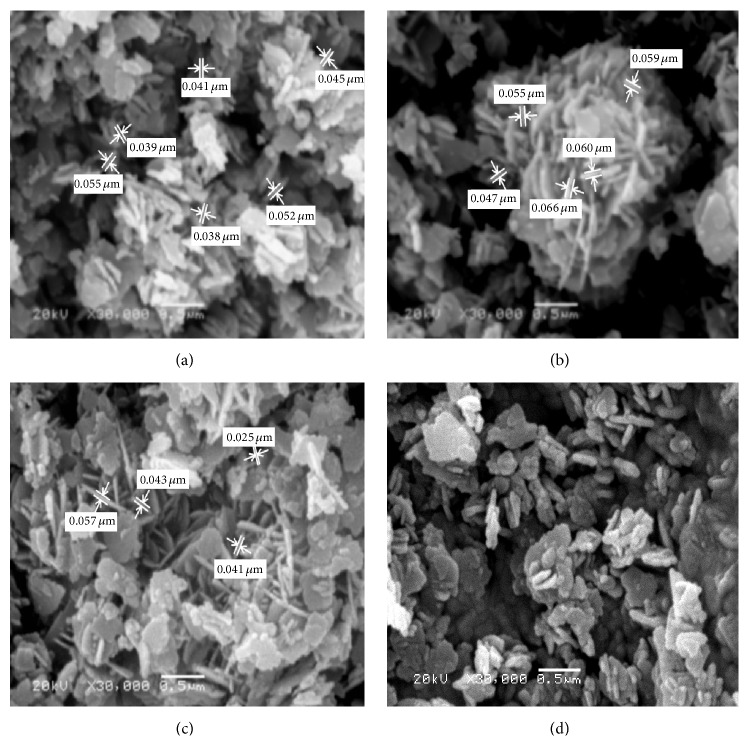
SEM micrographs of ZnO prepared using sol-gel technique at different reaction temperatures: (a) 60°C, (b) 70°C, (c) 80°C, and (d) 90°C.

*(ii) Morphological Structure (SEM).* The most proper selected surfactant (PVP) at different reaction temperatures (60, 70, 80, and 90°C) was tested for production of different zinc oxide configurations.  Figure 6 indicated that ZnO formed at either 60 or 80°C has nanoparticles configuration with average diameters ranging between 25 and 57 nm. However, those produced at 70°C have nanorods morphologies with average aspect ratio of 4. Also, nanoplates of ZnO have been produced at 90°C. This behavior may be attributed to the fact that the growth kinetic process of ZnO is slow at low temperatures, so the growth of nanospecies takes place at higher temperature to obtain different morphological structures. However, rise in reaction temperature results in increase in reaction rate, so ZnO grains rapidly grow, which leads to formation of nanorods with random orientations at 70°C. Also at higher temperature breaking rate of complexed metal ions releases more metal ions for rapid nucleation and growth process. The morphology evolution of ZnO as influence of temperature is due to nanorods getting squeezed together to form nanoparticles [33]. So, the most preferred temperature for production of uniform zinc oxide nanorods is 70°C. So, the reaction temperature has determinative effects on nano-zinc oxide formation [34].

It is generally believed that crystal formation of ZnO in solutions can be divided into two stages: crystal nucleation and growth. The effective reason for the reaction temperature affecting the morphology of ZnO could be attributed to different reaction pathways, solubility of the precursor, and rate of the reaction which influenced the crystal nucleation and growth [35]. This explains the case of ZnO production at 70°C, where at this case the growth rate dominates over the rate of nucleation which tends to form ZnO with nanorod structures (Figure 6(b)). However, the ZnO deformation mechanism was inverted for 60, 80, and 90°C where the nucleation rate is relatively higher than the rate of growth at lower and higher temperatures [36]. This creates great amounts of ZnO nuclei and limits the crystal growth rate, and then large-scale hexagonal ZnO particles were produced. At higher temperature, 90°C, nanoplates have been obtained. So, the most proper reaction temperature is that which produces uniform size distribution of ZnO nanorods with high aspect ratio of 70°C.

*(C) Effect of Reaction Time on Both the Crystalline and Morphological Structures of Prepared ZnO.* The time of the reaction solution is a key factor to the growth of nano-zinc oxide crystals. Therefore, time of the reaction mixture at the predetermined optimum conditions of zinc acetate precursor in the presence of PVP surfactant adjusting the reaction temperature at 70°C was varied in the range of 3, 6, 12, 24, and 48 hours. The structural and morphological properties are changed and identified with the help of XRD and SEM techniques.

**Figure 7:**
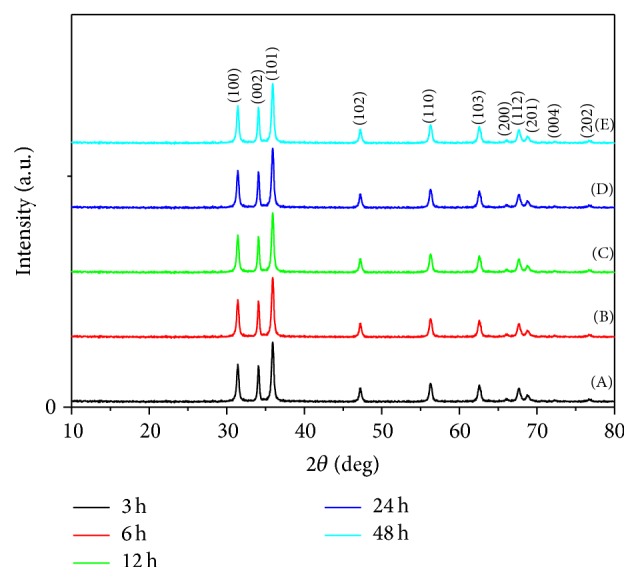
XRD patterns for ZnO nanopowders via sol-gel technique with different reaction times: (A) 3 h, (B) 6 h, (C) 12 h, (D) 24 h, and (E) 48 h.

*(i) Crystalline Structure (XRD).* Figure 7 showed XRD patterns for ZnO nanopowder that was prepared at different reaction time intervals (3, 6, 12, 24, and 48 hours). All the diffraction peaks can be indexed as the hexagonal wurtzite structure with high crystallinity. No impurity peaks have been detected from the XRD spectrum, where the entire crystalline precursors have decomposed and grew into ZnO single crystals [37]. Moreover, it is clear that the increase in the reaction time improves the degree of crystallinity of the formed ZnO [38].

**Figure 8:**
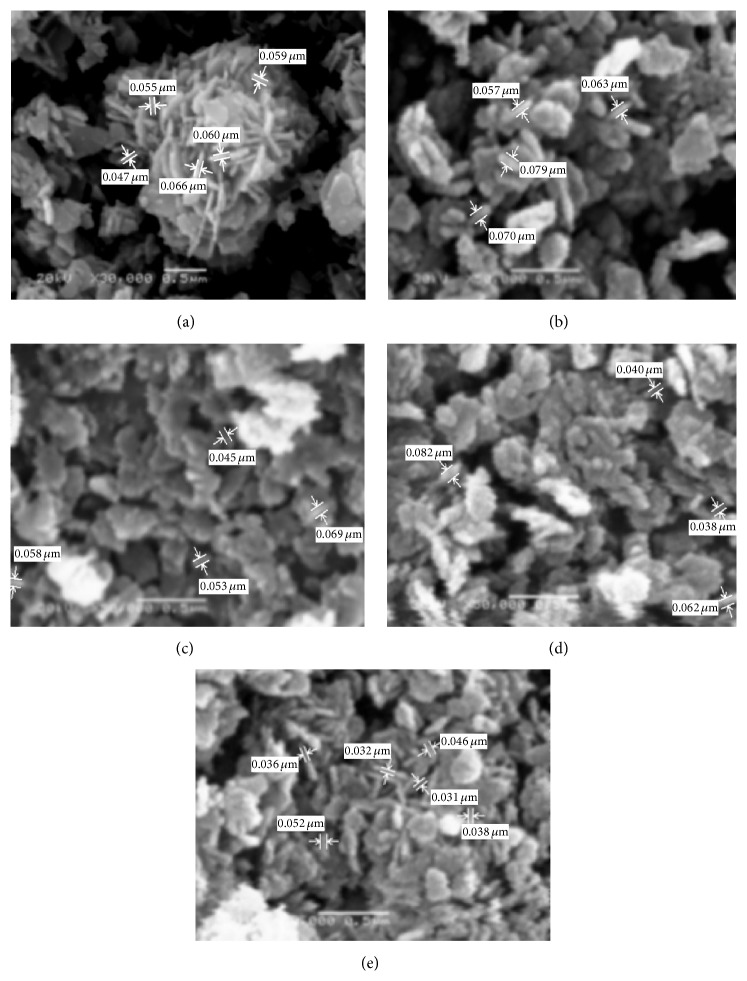
SEM micrographs of ZnO prepared using sol-gel technique with different reaction times: (a) 3 h, (b) 6 h, (c) 12 h, (d) 24 h, and (e) 48 h.

*(ii) Morphological Structure (SEM).* SEM images of the ZnO prepared at various reaction times were investigated in Figure 8. It is clear that the ZnO nanostructures depended on the growth time, where different morphological structures of nanoparticles and nanorods can be realized. For short growth periods of 3 hours (Figure 8(a)), aggregate nanorods structures are indicated. As the reaction period increased above 6 h, the nanorods disappeared and nanoparticles architecture morphology of ZnO can be achieved. However, for higher growth time, 6, 12, 24, and 48 h, nanoparticles structures have predominated. The suggested mechanism for ZnO may be considered as the ZnO nanorods structures are formed from the decomposition of Zn(OH)_2_ within 60 minutes' heating, after which the following time growth of the particles must result from dissolution and reprecipitation of the existing ZnO particles [39]. This is a slow process, as significant changes in the particle dimensions are only seen after 6 hours' ageing. As the reaction time increased above 6 hours, the formed nanorods structure of ZnO was converted to nanoparticles and nanoplates. That may be due to the increase in the stirring period of the formed ZnO nanoparticles after 6 h and destruction of the initial oriented growth chain of ZnO leading to particle formation from ZnO with hexagonal nanoparticle and nanoplates shapes. Consequently, the SEM results indicated that the optimum reaction time for ZnO nanorod formation with high degree of crystallinity and high aspect ratio was selected to be 3 hours.

### 3.2. Synthesis and Characterization of Nano-Zinc Oxide with Different Morphological Structures via Hydrothermal Technique

Hydrothermal technique has been used in nano-zinc oxide synthesis at constant pressure.

#### 3.2.1. Synthesis of Nano-Zinc Oxide in the Presence of Surfactant

*(1) Mechanism of Nano-Zinc Oxide Formation in the Presence of Surfactant.* In order to predict the formation mechanism involved in the hydrothermal process of ZnO synthesis in the presence of surfactant agent, the following chemical reactions may have been suggested:

(3) ZnCH3COO·2H2O+ZnOHn2−n⟶Zn2OOH2n−24−2n+H2O

(4) Zn2++2OH−⟶ZnOH2

(5) ZnOH2⟷ZnO+H2O

At the beginning of this process, reaction (3), where *n* = 2 or 4, was suspected to happen in solution; a Zn(OH)_2_ precipitate is formed [40]. So, the crystal structure of ZnO was constructed by reduction of Zn(OH)_2_ to form ZnO using surfactant. So, surfactant not only accelerates the reaction of the growth units, but also leads to their oriented growth, and the surface tension of solution is reduced, which decreases the energy needed to form a new phase [41].

*(A) Effect of Surfactant Type on Both the Crystalline and Morphological Structures of Prepared ZnO.* The different polyvinylpyrrolidone (PVP), polyvinyl alcohol (PVA), cetyltrimethylammonium bromide (CTAB), triethanolamine (TEA), and polyethylene glycol (PEG) weakly basic materials were tested as surfactant agents for ZnO production.

**Figure 9:**
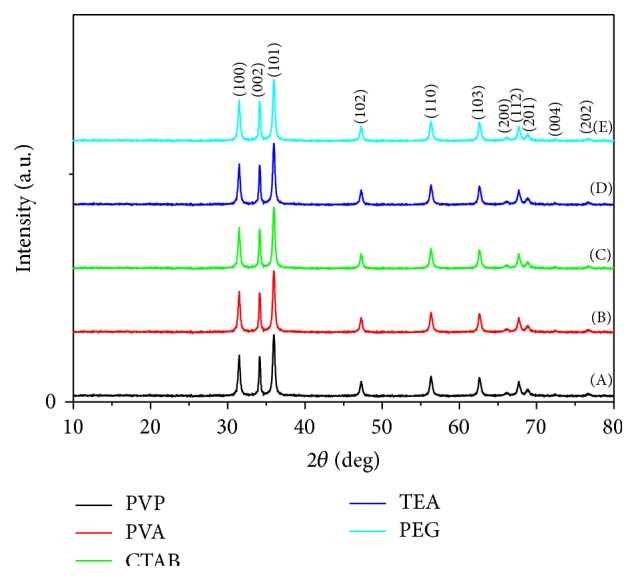
XRD patterns for ZnO nanopowders via hydrothermal technique with different surfactant agents: (A) PVP, (B) PVA, (C) CTAB, (D) TEA, and (E) PEG.

*(i) Crystalline Structure (XRD).* Figure 9 elucidates that the prepared ZnO samples using the different surfactant agents showed wurtzite crystal structure (hexagonal phase) with high intensity. The sharp peaks indicated that the products are well crystallized and oriented [22]. All the diffraction peaks can be well indexed to the hexagonal phase of ZnO reported in JCPDS card number 01-089-1397 and no other characteristic peaks are observed [23].

**Figure 10:**
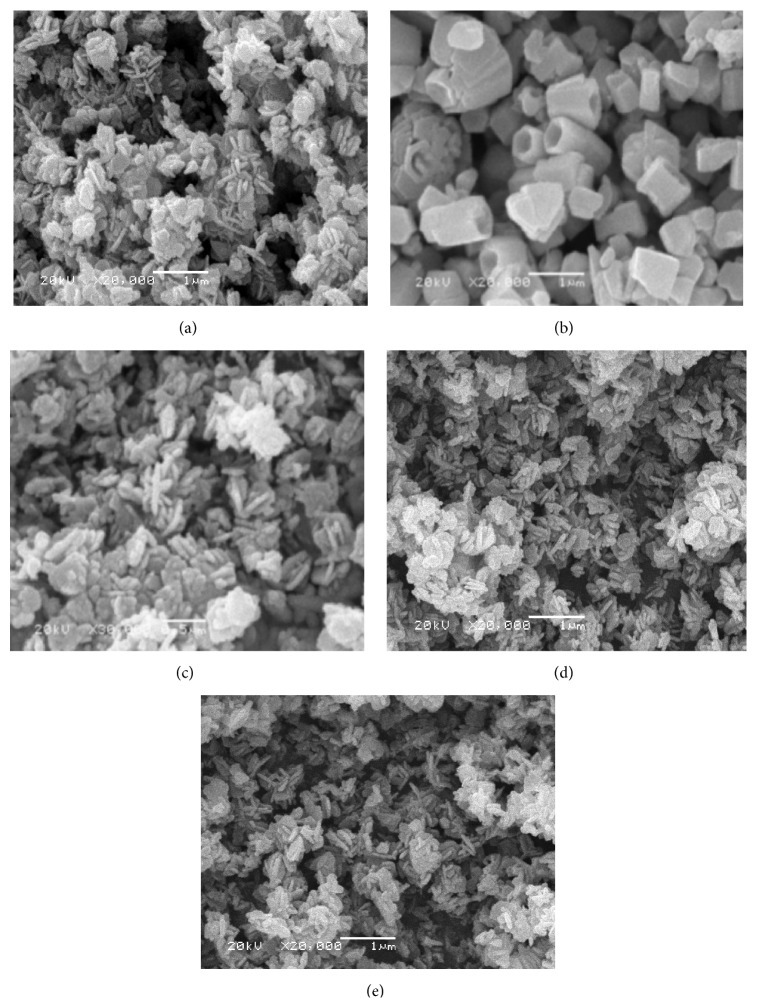
SEM micrographs of ZnO prepared using hydrothermal technique with different surfactant agents: (a) PVP, (b) PVA, (c) CTAB, (d) TEA, and (e) PEG.

*(ii) Morphological Structure (SEM).* The different attained zinc oxide morphological structures according to the surfactant variation are investigated in Figure 10. These images showed that ZnO morphologies were strongly dependent on the surfactant type in the hydrothermal process.  Figure 10(a) showed the formation of nanorods in the presence of PVP as surfactant. Also, hexagonal hollow tubes are formed using PVA surfactant. However, it is believed that the mechanism of ZnO nanotubes from nanowire-rod-like ZnO powders can be explained with the Kirkendall effect [42, 43]. In the Kirkendall effect, diffusion of atoms causes oversaturation of lattice voids. It is considered that this oversaturation causes condensation of more voids close to the interface. Therefore, these Kirkendall voids change properties of the interface and force it to form multiwalled nanotubes [28]. Furthermore, the presence of other studied surfactants (CTAB, TEA, and PEG) tends to form nanorods ZnO.

*(B) Effect of Reaction Temperature on Both the Crystalline and Morphological Structures of Prepared ZnO.* Regarding the great influence of the reaction temperature on the morphological structure of synthesized ZnO using sol-gel technique, the variation of this parameter will be monitored for the hydrothermal technique.

**Figure 11:**
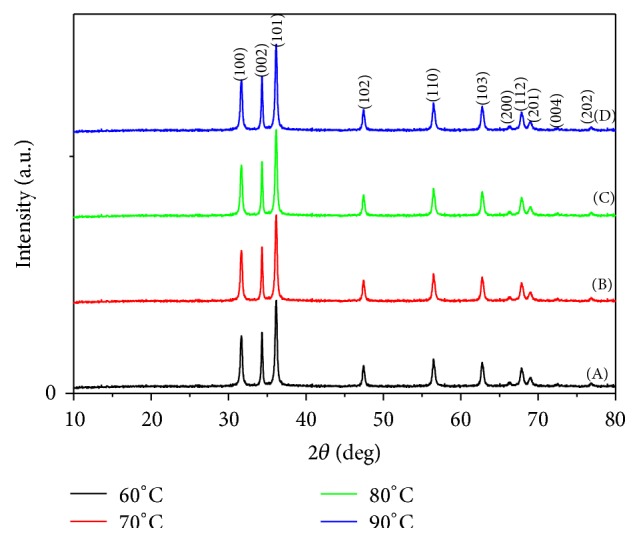
XRD patterns for ZnO nanopowders via hydrothermal technique at different reaction temperatures: (a) 60°C, (b) 70°C, (c) 80°C, and (d) 90°C.

*(i) Crystalline Structure (XRD).* It was proved that the formation of ZnO powder depends on the reaction temperature [33]. In order to determine the drawback of the formed ZnO in its crystallinity, XRD patterns of the ZnO nanopowder prepared under different temperatures (60, 70, 80, and 90°C) are shown in Figure 11 [32]. All the diffraction peaks can be well indexed to the hexagonal phase of ZnO reported in JCPDS card number 01-089-1397 and no obvious characteristic peaks are observed for other impurities.

**Figure 12:**
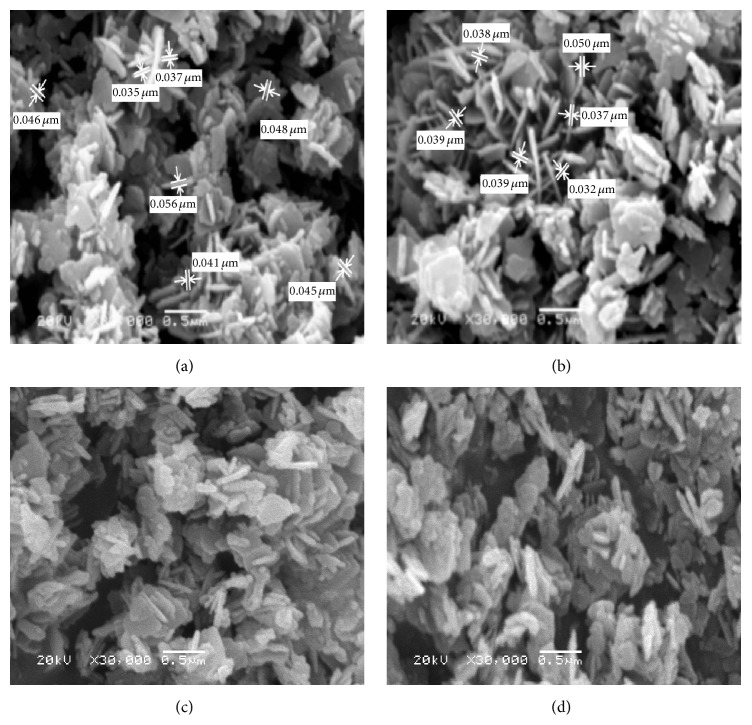
SEM micrographs of ZnO prepared using hydrothermal technique at different reaction temperatures: (a) 60°C, (b) 70°C, (c) 80°C, and (d) 90°C.

*(ii) Morphological Structure (SEM).* Figure 12 illustrated the surface morphology of nano-zinc oxide produced at different reaction temperatures. It is observed that ZnO formed at 60, 70, and 80°C has nanorods configuration with aspect ratio 6 (Figures 12(a), 12(b), and 12(c)); however, that produced at 90°C has nanoplates morphology. Nevertheless, with lower growth temperature below 90°C, the formed ZnO produced nanorods. This may be owing to the decrease in the growth rate. Where, at low temperatures, the growth kinetic tends to be slow, two-dimensional (2D) growth of nanospecies takes place which leads to formation of rod morphology. However, the rise in temperature increases the reaction rate, so ZnO grains rapidly grow, which leads to formation of hexagonal particles and belts with random orientations [33].

It is generally believed that the effective reason for the reaction temperature affecting the morphology of ZnO could be attributed to different reaction pathways, solubility of the precursor, and rate of the reaction which influenced the crystal nucleation and growth [44]. It is concluded from the previous results of ZnO morphology that lower temperatures produced nanorods structure where higher temperatures produced nanobelts. So, reaction temperature of 70°C was selected as the optimum temperature.

*(C) Effect of Reaction Time on Both the Crystalline and Morphological Structures of Prepared ZnO.* The reaction time of the reaction mixture at the predetermined optimum conditions in the presence of PVP surfactant using sodium hydroxide solution at 70°C and constant pressure was varied in the range of 3, 6, 12, 24, and 48 hours. The structural and morphological properties are changed and identified.

**Figure 13:**
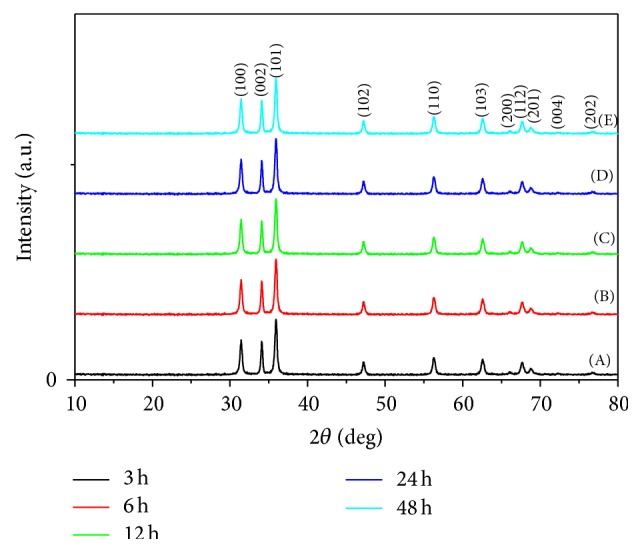
XRD patterns for ZnO nanopowders via hydrothermal technique with different reaction times: (A) 3 h, (B) 6 h, (C) 12 h, (D) 24 h, and (E) 48 h.

*(i) X-Ray Diffraction Analyses.* The XRD patterns of five different times of reaction were obtained as shown in Figure 13. All the diffraction peaks could be indexed to hexagonal wurtzite ZnO (JCPDS card number 01-089-1397) with high crystallization. No characteristic peaks were observed for other impurities such as Zn or Zn(OH)_2_ [45]. The increase in the reaction time improves the degree of crystallinity of the formed ZnO. These results are compatible with outcome of McBride and others [41], who have studied the effect of reaction time on zinc oxide crystalline structure using the aqueous chemical growth technique.

**Figure 14:**
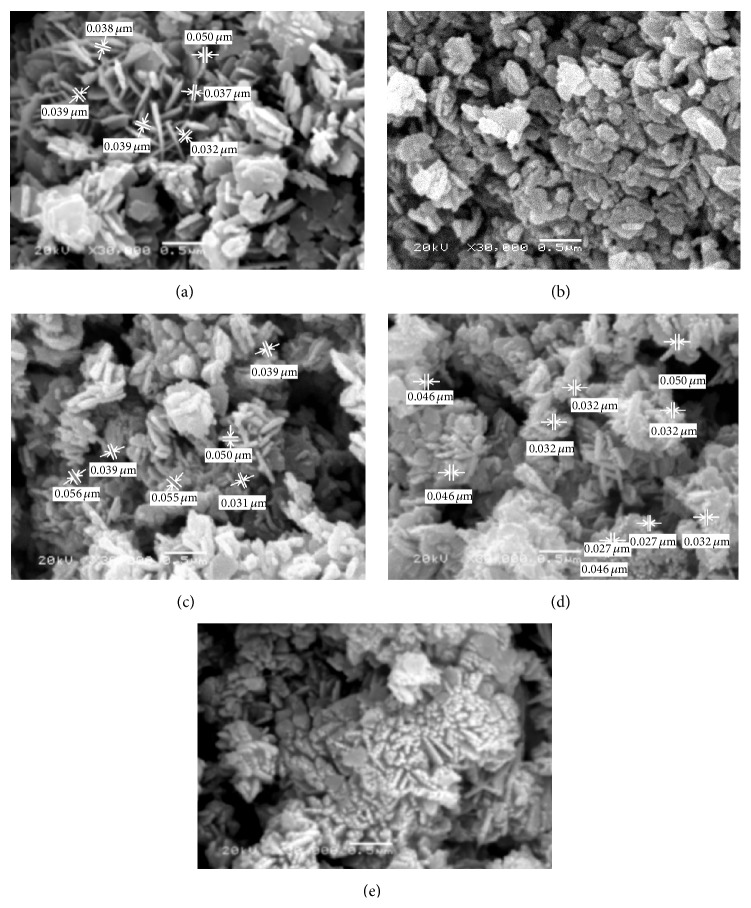
SEM micrographs of ZnO prepared using hydrothermal technique with different reaction times: (a) 3 h, (b) 6 h, (c) 12 h, (d) 24 h, and (e) 48 h.

*(ii) Morphological Structure (SEM).* Figure 14 illustrated that ZnO nanostructures depended on the growth time for the hydrothermal technique, where different morphological structures of nanorods, nanoplates, and nanoparticles can be seen. For short growth periods of 3 hours (Figure 14(a)), aggregate nanorods structures are indicated. As the reaction period increased at 6 h, the nanorods disappeared and nanoplates architecture morphology of ZnO can be achieved. However, for higher growth time above 6 h, nanoparticles structures have predominated.

### 3.3. Influence of Annealing Process on Zinc Oxide Physical Properties

As all known and expected, the prepared zinc oxide nanotube produced from the hydrothermal technique in the presence of PVA as stabilizing agent will attain the highest surface area value compared with the other prepared architecture morphology of ZnO. Accordingly, this sample was assigned as the most proper prepared zinc oxide sample to test the influence of the annealing process on the material crystallinity, morphology, and surface area.

**Figure 15:**
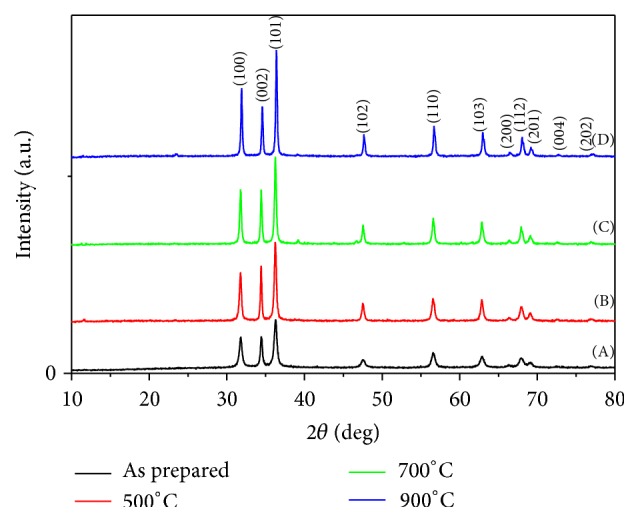
XRD patterns of nanotube ZnO after and before annealing at different temperatures: (A) as prepared, (B) 500°C annealing, (C) 700°C annealing, and (D) 900°C annealing.

*(i) Crystalline Structure (XRD).* Figure 15 investigated the influence of the annealing temperature on zinc oxide degree of crystallinity. It was explored from the comparable XRD patterns that the as-prepared nanotube ZnO attains the lowest crystallinity degree compared with the different annealed samples. Moreover, it was elucidated that, as the annealing temperature increased, the diffraction characteristics peaks of ZnO became narrower and exhibit a higher intensity, which further suggests that the nanotubes become more crystalline which may change the material morphology [46].

**Figure 16:**
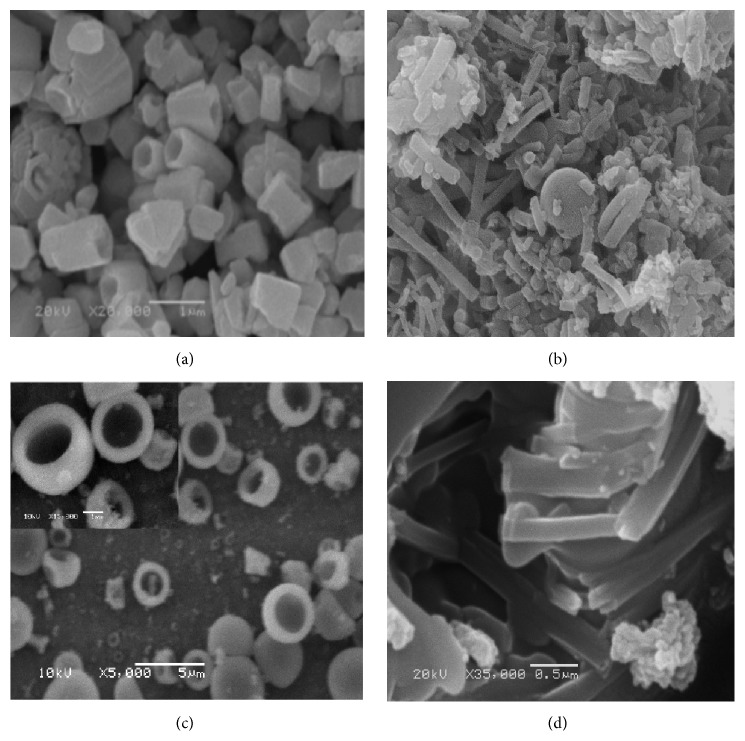
SEM micrographs of ZnO nanotubes after and before annealing at different temperatures: (a) as prepared, (b) 500°C annealing, (c) 700°C annealing, and (d) 900°C annealing.

*(ii) Morphological Structure (SEM).* In order to elucidate the effect of annealing temperature on the zinc oxide morphological structure, the structure of the most proper prepared sample in nanotube morphology was compared with the different structures after annealing the sample at three various temperatures. It was indicated from Figure 16 that the annealing temperature has significant impact on the ZnO morphological structure as expected from XRD results. However, the annealing of nanotube sample at 500°C improves the orientation degree of ZnO nanotubes compared with the as-prepared sample. However, as the annealing temperature improved up to 700°C, the morphological structure of ZnO was converted completely into cup-like structure with average diameter of 0.5 micrometers. This conversion at the morphological structure may be regarded as the microwave annealing process of ZnO that enhances the grain-growth rate and increases the densification behavior of the materials that yield the microcups structures [47]. At the highest studied annealing temperature of 900°C, the nanotube morphology of ZnO tends to aggregate to form aggregated tubes that may be attributed to the high annealing temperature that may break down the nanotubes boundaries to yield aggregated microtube morphological structure [48].

*(iii) Surface Area (BET).* With respect to the high impact of the microwave assisted annealing process on zinc oxide crystallinity and morphological structures, the influence of the annealing process on the material surface area and its pore size was tabulated in Table 1. It was investigated from this table that the annealing process decreases the zinc oxide surface area and its pore size. This may be attributed to sintering impact of the annealing temperature on the material that dissolving the materials boundaries and form large aggregates from nanoscale into microscale dimensions that by its role dramatically decrease the material surface area and its pore size. Moreover, it was evident from Table 1 that the as-prepared nanotube ZnO has high surface area and pore size slightly affected after microwave annealing at 500°C. The depression at the material surface area was noticed after annealing at 700°C, where at this temperature the material surface area decreased from 17.8 to 7.2 m^2^/g. These results give prediction that the annealed ZnO samples will have less antimicrobial activity compared with the as-prepared nanotube ZnO sample.

### 3.4. Antimicrobial Activity of Nanotubes Zinc Oxide

**Figure 17:**
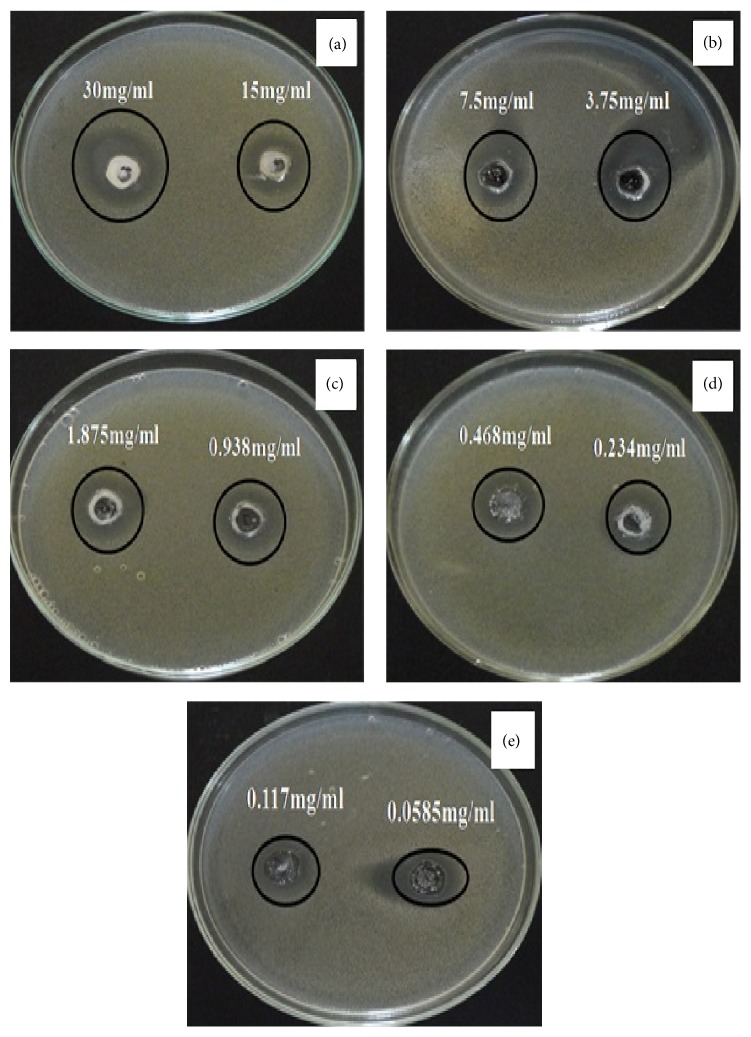
The antimicrobial activity of zinc oxide nanotubes against* Escherichia coli* using disc diffusion method. ((a), (b), (c), (d), and (e)) Different concentrations of the synthesized ZnO.

Regarding the high surface area of the as-prepared zinc oxide nanotube produced from the hydrothermal technique in the presence of PVA as stabilizing agent which was equivalent to 17.8 m^2^/g, its bactericidal activity was tested* in vitro* against four standard bacterial isolates: Gram-positive (*Staphylococcus aureus *and* Bacillus subtilis)* and two Gram-negative (*Escherichia coli* and* Pseudomonas aeruginosa*) strains.  Table 2 and Figure 17 indicate that* Escherichia coli* was highly affected by nano-zinc oxide where the growth inhibition reached 32 and 29 mm using 30 and 15 mg/mL of nano-zinc oxide, respectively. The presence of an inhibition zone clearly indicated the antibacterial activity of zinc oxide nanotubes. However, by increasing the concentration of nano-ZnO in discs, the inhibition zone has also been increased [49]. The minimum inhibitory concentration (MIC) from the synthesized nano-ZnO was 0.0585 mg/mL. This recorded MIC value is very small compared with that published in another research of 3.4 mg/mL for antibacterial properties of ZnO nanoparticles tested on* Escherichia coli *[49].

**Figure 18:**
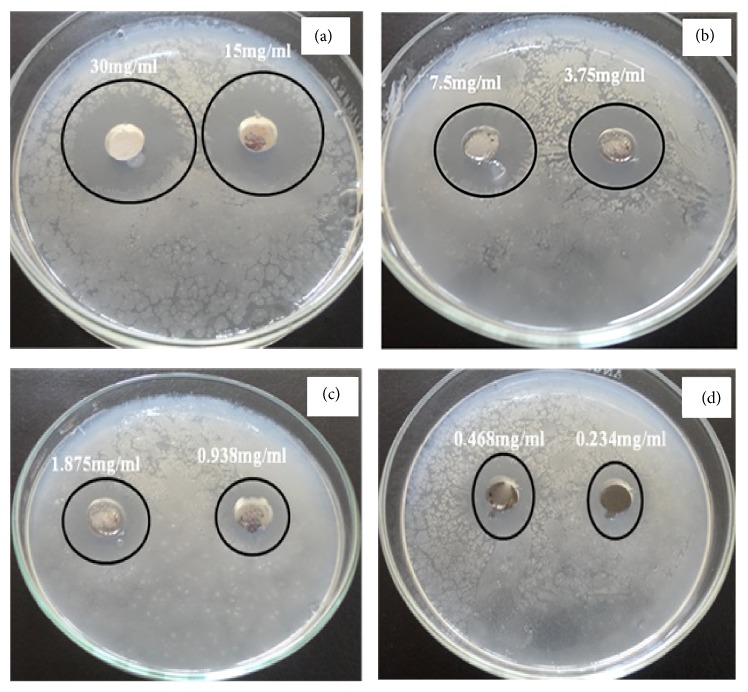
The antimicrobial activity of zinc oxide nanotubes against* Pseudomonas aeruginosa* using disc diffusion method. ((a), (b), (c), and (d)) Different concentrations of the synthesized ZnO.

The antibacterial efficiency of ZnO nanotubes was also investigated against* Pseudomonas aeruginosa*. The results reveal a high activity of the obtained nano-zinc oxide and the minimum inhibitory concentration (MIC) was 0.234 mg/mL as illustrated in Table 2. From Figure 18, it was observed that* Pseudomonas aeruginosa* was highly affected by nano-zinc oxide where the growth inhibition ranged between 37 and 32 mm with zinc oxide suspension concentration of 30 and 15 mg/mL, respectively. These results indicated that zinc oxide nanotubes had antibacterial effects on* Pseudomonas aeruginosa*, which is partly in accordance with the previous reports [44] on the antibacterial properties of nano-ZnO.

**Figure 19:**
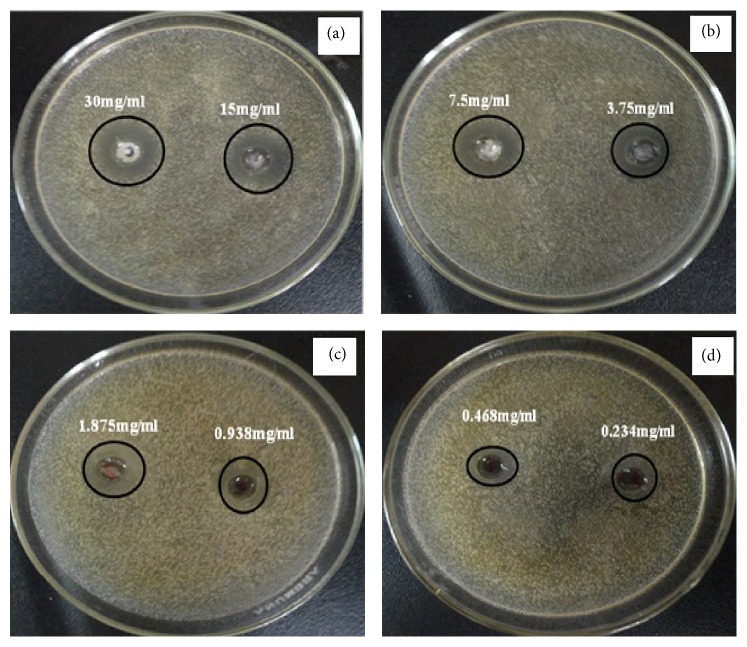
The antimicrobial activity of zinc oxide nanotubes against* Staphylococcus aureus* using disc diffusion method. ((a), (b), (c), and (d)) Different concentrations of the synthesized ZnO.

Antimicrobial activity of ZnO nanotubes against* Staphylococcus aureus* is illustrated in Figure 19 and its inhibition zones (mm) were tabulated in Table 2, indicating that zinc oxide nanotubes have moderate activity (24 and 22 mm) against* Staphylococcus aureus *when concentrations 30 and 15 mg/mL were used [44, 45].

**Figure 20:**
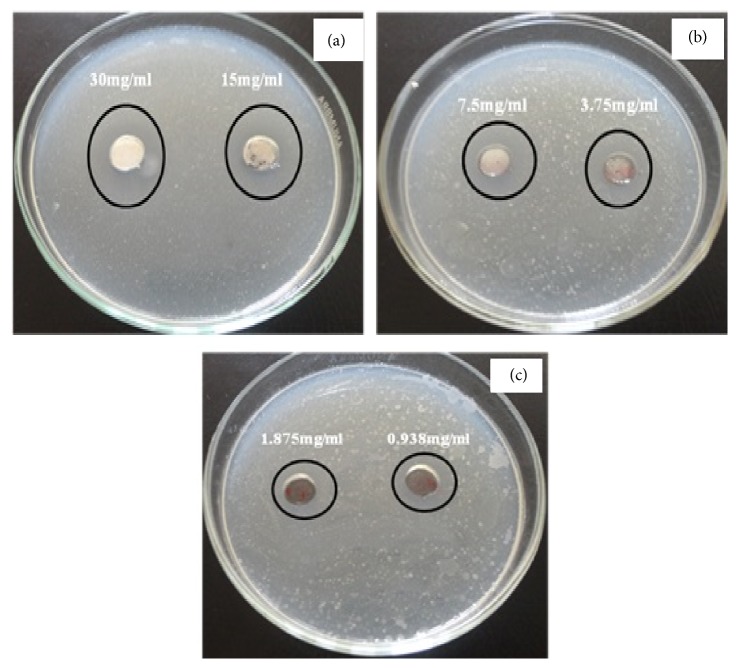
The antimicrobial activity of zinc oxide nanotubes against* Bacillus subtilis* using disc diffusion method. ((a), (b), and (c)) Different concentrations of the synthesized ZnO.

Figure 20 investigated the activity of nanotube zinc oxide against* Bacillus subtilis *using the disc diffusion method. The improvement at the inhibition zones from 21 mm to 23 mm was observed as nanotube zinc oxide concentration increased from 15 mg/mL to 30 mg/mL. The synergetic effect of the zinc oxide nanotubes was observed by the increase in diameter of inhibition zones (mm) which has been recorded in Table 2. These results agreed well with the growth inhibition demonstrated against zinc oxide nanoparticles [49].

Accordingly, it was established from these results that the* Bacillus subtilis* strain represents stronger zinc oxide antiresistance bacterial isolates.

Based on all MICs obtained by the different synthesized ZnO, our synthesized ZnO nanotubes have high MIC, which makes them candidate to be used as antibacterial in different applications. On the other hand, from the results obtained in disc agar diffusion method, it can be suggested that, in comparison with Gram-negative bacteria, the growth of Gram-positive bacteria is inhibited at higher concentrations of ZnO nanotubes. According to the results, it can be concluded that zinc oxide nanotubes are effective antibacterial agents both on Gram-positive and Gram-negative bacteria.

### 3.5. Influence of Annealing Process on Zinc Oxide Antimicrobial Activity

**Figure 21:**
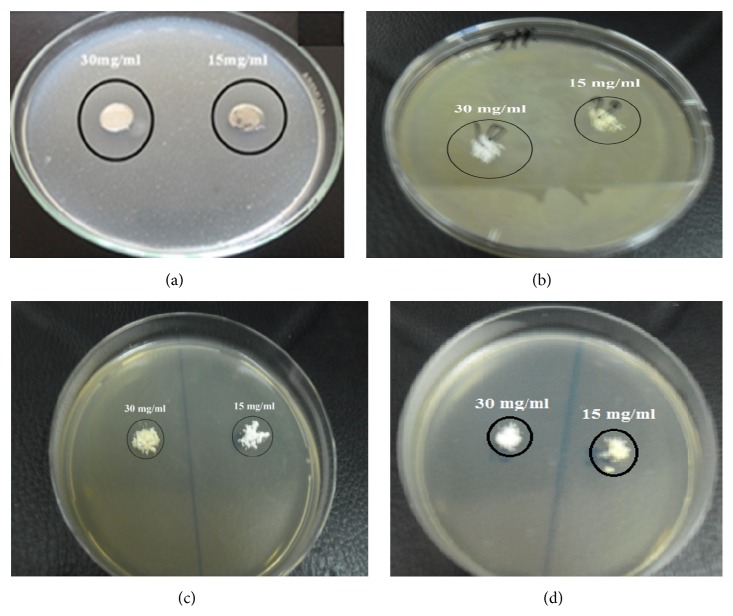
The influence of annealing process of zinc oxide on its antimicrobial activity against* Bacillus subtilis* using disc diffusion method. (a) As-prepared nanotube ZnO, (b) annealed ZnO at 500°C, (c) annealed ZnO at 700°C, and (d) annealed ZnO at 900°C.

**Figure 22:**
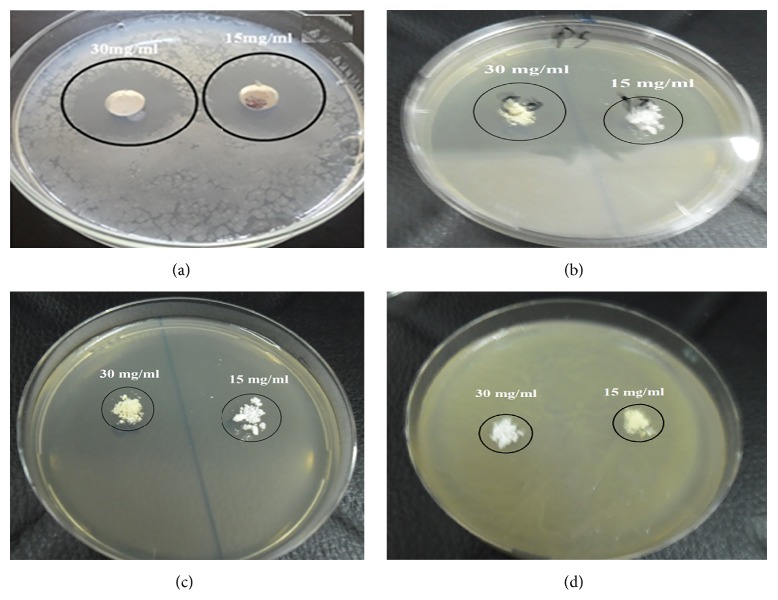
The influence of annealing process of zinc oxide on its antimicrobial activity against* Pseudomonas aeruginosa* using disc diffusion method. (a) As-prepared nanotube ZnO, (b) annealed ZnO at 500°C, (c) annealed ZnO at 700°C, and (d) annealed ZnO at 900°C.

In order to elucidate the influence of the microwave annealing process of nanotube zinc oxide on antimicrobial activity, the annealed ZnO samples were examined against the Gram-positive (*Bacillus subtilis*) and Gram-negative (*Pseudomonas aeruginosa*) isolates at the optimum predetermined ZnO concentrations of 15 and 30 mg/mL. It was explored from Figures 21 and 22 that the annealing process of ZnO has negative impact on its antimicrobial activity for both Gram-positive and Gram-negative bacteria.  Figure 21 indicated that the inhibition zones of ZnO against* Bacillus subtilis* decreased from ~22 mm for the as-prepared ZnO nanotubes to ~14 mm for the annealed sample at 500°C and these inhibition zones tend to be less than 5 mm for the annealed samples at 900°C. With respect to the Gram-negative strain (*Pseudomonas aeruginosa)*, Figure 22 evidenced that the inhibitions zones decline from ~35 mm for the as-prepared nanotubes to ~21 mm for the annealed sample at 500°C and these inhibition zones tend to decline less than 10 mm for the annealed samples at 900°C. The depression at the inhibition zones of ZnO annealed samples compared with the as-prepared nanotube ZnO sample may be attributed to the decline at the material surface area after annealing which was proved previously as the action of the annealing temperature.

## 4. Conclusion 

Nano-zinc oxide with different morphological structures was successfully synthesized using two different techniques. The samples were examined using X-ray diffraction (XRD) and scanning electron microscopy (SEM) to be sure that they are in nanoscale with different morphological structures (particles, rods, and tubes). The surfactant has a significant role in both the rate of nucleation and growth mechanisms which are responsible for the structure of nano-ZnO formed. The improvement in the reaction temperature enhances both the reaction rate and the degree of ZnO crystallinity. The ZnO morphological structure was strongly dependent on the reaction time. The synthesized zinc oxide nanotubes have a strong antimicrobial activity against some Gram-positive and Gram-negative bacteria. This antimicrobial activity was decreased with increasing annealing temperature of ZnO from 500 to 900°C. Accordingly, the synthesized ZnO nanotubes can be utilized as nanocontrol materials against the medicinal bacteria. Gram-positive bacteria seemed to be more resistant to zinc oxide nanotubes compared with Gram-negative bacteria. It was found that the antibacterial activity of zinc oxide nanotubes increased with increasing powder concentration.

## Figures and Tables

**Table 1:** Effect of annealing temperature on ZnO surface area and pore size.

ZnO sample	Surface area, m^2^/g	Average pore size, nm
As-prepared nanotube (hydrothermal technique)	17.8	278.6
500°C annealing	16.7	255.4
700°C annealing	7.2	114.6
900°C annealing	2.4	53

**Table 2:** The antimicrobial activity as measurable inhibition zones against different bacterial strains using various concentrations of ZnO nanotubes suspensions.

ZnO concentration (mg/mL)	Zinc oxide inhibition zones (mm)			
	*Escherichia coli *	*Pseudomonas aeruginosa *	*Staphylococcus aureus*	*Bacillus subtilis*
30	32	37	24	23
15	29	32	22	21
7.5	27	28	21	19
3.75	26	27	20	18
1.875	23	26	19	17
0.938	22	24	18	15
0.468	21	21	16	0
0.234	19	18	14	0
0.117	17	0	0	0
0.0585	13	0	0	0
0.02925	0	0	0	0
